# Influence of Different Activators on the Structure and Properties of Activated Carbon Based on Bamboo Fiber

**DOI:** 10.3390/polym14245500

**Published:** 2022-12-15

**Authors:** Peng Lin, Yao Xia, Zhigao Liu

**Affiliations:** School of Resources, Environment and Materials, Guangxi University, Nanning 530004, China

**Keywords:** bamboo fiber-based, activated carbon, electrochemical properties, methylene blue adsorption

## Abstract

In order to explore the influence of different activators on the structure and properties of the prepared activated carbon, bamboo fiber-based activated carbons (BFACs) were prepared by four activators of phosphoric acid, pyrophosphoric acid, zinc chloride, and diammonium biphosphate (BFAC-H_3_PO_4_, BFAC-H_4_P_2_O_7_, BFAC-ZnCl_2_, and BFAC-(NH_4_)_2_HPO_4_) and BFACs adsorption performance and electrochemical properties were investigated. The main conclusions were: the specific surface area of the four BFACs varies greatly, among which BFAC-ZnCl_2_ was the highest, at 1908.5074 m^2^/g, and BFAC-(NH_4_)_2_HPO_4_ was the lowest, at 641.5941 m^2^/g. In terms of the pore structure, BFAC-H_3_PO_4_ and BFAC-H_4_P_2_O_7_ are mainly mesopores and BFAC-ZnCl_2_ and BFAC-(NH_4_)_2_HPO_4_ are mainly micropores. The BFAC-ZnCl_2_ sample had the largest specific capacitance, with a specific capacitance of 121.2730 F/g at a current density of 0.2 A/g, with a small internal resistance and good electrochemical reversibility and capacitance performance. The adsorption properties were better for BFAC-ZnCl_2_ and BFAC-H_3_PO_4_ and the adsorption amounts were 648.75 and 548.75 mg/g, respectively.

## 1. Introduction

In recent years, wastewater treatment is becoming increasingly important as global warming worsens, freshwater resources decrease, and environmental problems become more and more serious. The removal of contaminants from wastewaters is a major challenge in the field of water pollution. Among numerous techniques available for contaminant removal, adsorption using solid materials and named adsorbents is a simple, useful, and effective process [1]. Among the sustainable high-performance electrochemical energy storage technologies, supercapacitors have drawn substantial attention due to their superior power density, ultra-fast charge–discharge rate, high reversibility, long cycle life, and relatively low cost. Nonetheless, the main obstacle presented by supercapacitors is their relatively low energy–density values compared to batteries [2].

Activated carbon, formed by the accumulation of six rings of carbon, is an excellent adsorbent material with a rich internal pore structure and specific surface area. The adsorption capacity of activated carbon depends on its physical and chemical properties such as pore size, pore capacity, specific surface area, and chemical functional groups. In general, the larger the specific surface area, the higher the adsorption capacity of activated carbon [3]. Nowadays, activated carbon has become increasingly popular among scientists because of its simple preparation process and abundant and inexpensive raw materials [4] and it is widely used in industry, agriculture, life, medicine, and health, environmental protection, electronics, and military fields. With the development of carbon material preparation technology, the application of bamboo charcoal and bamboo-based activated carbon will be more promising [5]. The structure and properties of activated carbon are affected by the raw material and activation process, so it has become more and more necessary to explore the preparation of activated carbon from biomass.

Biomass is natural raw material for activated carbon as it is usually loose in structure and has well-developed micro-pores during combustion and pyrolysis [6]. In addition, as a renewable resource widely found in nature, biomass has the advantages of being diverse, low cost, environmentally friendly, easily accessible, and abundant [7]. At present, the biomass commonly used in activated carbon raw materials includes crop straw, fruit shells, wood, and bamboo or bamboo processing waste. However, there is less research on the refinement of raw materials and the high value that can increase environmental benefits. As an important biomass resource in China, bamboo has the advantages of short growth cycle, strong renewability, abundant resources, and large accumulation and is known as the “second forest” in China [8]. The chemical composition of bamboo is similar to that of wood, consisting mainly of cellulose, lignin, and hemicellulose, with the highest cellulose content of about 55%, lignin about 25%, and hemicellulose about 20%. The tissue structure of bamboo is relatively simple and is mainly composed of parenchyma cells (PCs) and fiber cells (FCs). The hollow ultra-high aspect ratio of FCs can be considered the natural long one-dimensional fibers with electron transfer potential [9]. The content of C and O elements within bamboo fiber is high and the microfibrillar skeleton of bamboo fiber is more likely to form a pore structure, which is a good activation green precursor.

The following is the research progress of activated carbon in China and abroad. Tran et al. reported an activated carbon created using coffee husk with a high adsorption capacity for pollutants, which was activated using KOH agents [10]. The advantage of activation in the KOH solution was the simultaneous combination of chemical activation and pyrolysis. However, it consumes a lot of chemicals and cannot reuse residual chemicals after activation, resulting in the increase in total cost. In an article by Chaparro-Garnica et al., hemp residue-based activated carbons were prepared using H_3_PO_4_-assisted hydrothermal carbonization using a low concentration of H_3_PO_4_ [11]. This activation method had a lower polluting effect compared to other chemical activating agents. Moreover, H_3_PO_4_ can be reused after recovering it during the washing step. However, the main disadvantage of H_3_PO_4_ activation lies in increasing the cost of production as a consequence of a large number of washing steps. In the research of Mohammad et al., activated carbon was synthesized from Jatropha curcas L. seed hull through chemical activation with ZnCl_2_ [12]. Zinc chloride (ZnCl_2_) had several reported advantages toward physical activation such as it can improve the pore creation and provides higher yield and increases the BET surface area. However, the use of ZnCl_2_ as the activation agent had environmental disadvantages due to zinc chloride’s high corrosivity. Hekmatshoar et al. reported a NH_4_Cl-induced activated carbon was synthesized by a simple method and used for the degradation of PG in contaminated water [13]. The granules were impregnated by NH_4_Cl to increase the porosity and thereby the adsorption capacity of carbon while activating an ammonium explosion. Therefore, it is of great practical significance to study the effect of different activators on activated carbon’s physical and chemical properties.

Selectively removing lignin and/or hemicelluloses, thus reducing energy and resource consumption, is ideal for functionalized biomass [14]. The research and preparation of lignin-based activated carbon began in the 1970s and 1980s, when Japanese and Soviet scientists studied the preparation of activated carbon with lignin carbonization as the precursor. At present, some scholars also prepare activated carbon from lignin as raw material. Fu et al. prepared steam physically activated carbon using black liquor lignin obtained from the pulp and paper industry [15]. However, the activated carbon prepared with lignin is mostly in powder form, which is easy to lose when used, difficult to recycle, and difficult to transport and store, so it is usually necessary to add a binder during preparation, resulting in complicated processes. With the improvement of China’s economy, science and technology, and people’s living standards, the demand for products that can improve the living and working environment and benefit environmental health is increasing and granular activated carbon has overcome the shortcomings of powdered activated carbon, so it will receive more and more attention and application in the field of adsorption purification [16].

In this work, bamboo fiber-based activated carbon was prepared by removing lignin and using the chemical activation method; the effects of different activators on the physicochemical properties of activated carbon are discussed. Our report revealed the inevitable connection between the activators and the activated carbon structure. The effect of activated carbon structure on the electrochemical performance and dye adsorption performance was also analyzed.

## 2. Experimental

### 2.1. Materials and Instruments

The bamboo raw material used in this paper was obtained from natural southern bamboo produced in the Fuyang District, Hangzhou, China. A DF-101S collector type constant temperature heating magnetic stirrer, MFLGKDF406-12 muffle furnace, PHS-2F type PH meter, Nicolet IS Model 50 Fourier Transform Infrared Spectrometer, Mike ASAP2460 fully automatic specific surface and porosity analyzer BET, and UV-5500 UV-Vis spectrophotometer were the instruments used in this paper.

### 2.2. Activated Carbon Preparation

The raw material was first delignified by impregnating the bamboo strips with a solution of sodium chlorite (NaClO_2_), heating them in a water bath at 80 °C for 6 h. After 6 h, the solution was replaced and the heating was continued, repeating the above steps 8 times until the bamboo strips turned white. Finally, the white bamboo strips were dried in a freeze-drying oven and separated to obtain bamboo fibers. A certain amount of bamboo fiber was mixed with four activators (phosphoric acid, pyrophosphoric acid, zinc chloride, and diammonium biphosphate) at an impregnation ratio of 3:1 and stirred well, dried, and transferred to a tube furnace and activated at 600 °C for 90 min. The impregnation ratio, activation temperature, and activation time were all selected to fully activate the bamboo fiber. Finally, the obtained activated carbon was washed repeatedly until the pH of the aqueous solution was 6–7. The four prepared activated carbon samples were named as BFAC-H_3_PO_4_, BFAC-H_4_P_2_O_7_, BFAC-ZnCl_2_, and BFAC-(NH_4_)_2_HPO_4_.

### 2.3. Characterization

The pore structures of the activated carbon samples were obtained using nitrogen adsorption and desorption tests. The total surface area of the activated carbon was calculated using the BET method. The specific surface area and pore volume of the micropores and mesopores were obtained using the t-curve method and the Barrett-Joyner-Halenda method, respectively. The pore size distribution was calculated using the Nonlocal Density Functional Theory method. The functional groups of activated carbon were analyzed using infrared spectroscopy. The high purity potassium bromide and dried activated carbon samples were thoroughly ground in a 100:1 ratio in a natural agate mortar and then pressed into transparent tablets using a tablet press and tested in an infrared spectrometer with a scanning range of 500–4000 cm^−1^. In this paper, the crystal composition of the prepared activated carbon samples was analyzed using X-ray diffraction (XRD) with a Rigaku D/MAX 2500 V model (Rigaku, Japan) with a scanning range from 5 to 65° and a scanning speed of 10°/min. Finally, the surface elements and valence states of the activated carbon samples were characterized using X-ray photoelectron spectroscopy (XPS).

### 2.4. Electrochemical Performance Test

A certain amount of PTFE, acetylene black, and activated carbon samples (ratio 1:1:8) were weighed in a natural agate body mortar, thoroughly polished and stirred well before being placed on a nickel sheet 2 cm long and 1 cm wide, then wrapped with a film of electrode material and squeezed with a pneumatic press to obtain an electrode sheet. Subsequently, the electrode sheet was placed in an uncovered glass container and transferred to a vacuum drying chamber for evacuation, with the temperature set at 80 °C for 12 h. The electrode sheet was placed in an uncovered glass container and impregnated with a 6 M potassium hydroxide solution, ensuring that the solution does not pass through the electrode sheets and then transferred to the vacuum drying chamber with the heating procedure switched off and vacuum evacuation only for 8 h to allow for the complete penetration of the electrode sheets. The electrode sheet was then removed, assembled with the three electrodes, and tested by adding 6 M potassium hydroxide and 1 M potassium hydroxide solution to the electrolytic cell and reference electrode. Finally, the Faraday EM shield was connected to an electrochemical workstation and linear cyclic voltammetry, constant current charge/discharge, and impedance sweep tests were carried out using CS Studio5 software.

### 2.5. Adsorption Performance Test

**Figure 1:**
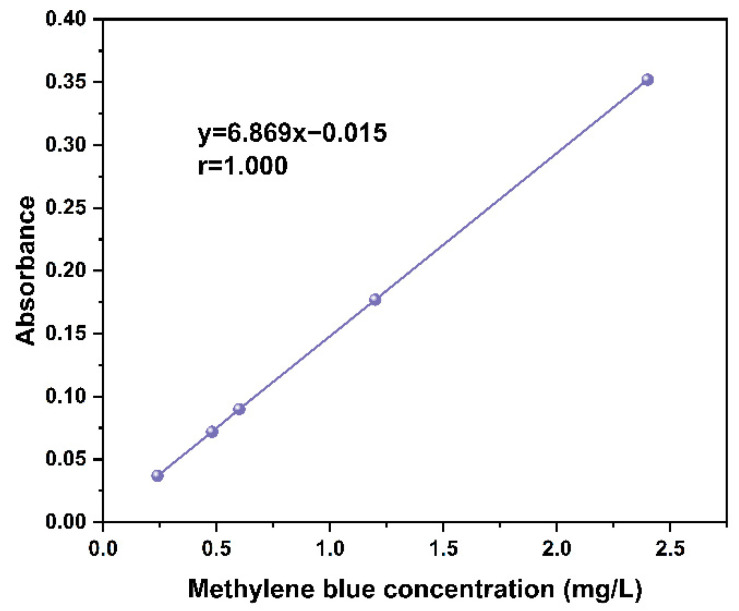
Standard curve of methylene blue.

Next, 3.6 g of potassium dihydrogen phosphate and 14.3 g of disodium hydrogen phosphate were dissolved in 1000 mL of water and then the buffer solution was heated in a water bath at 60 °C, at which the pH of the buffer solution was about 7. The actual content of the 1.5 g of methylene blue sample was dissolved in the water bath heated buffer solution, stirred well until fully dissolved, cooled to room temperature, and filtered into a 1000 mL volumetric flask; the concentration of the buffer solution was 1.5 g/L. Moreover, 100 mL of the above solution was weighed into a 500 mL volumetric flask and diluted with a buffer solution to configure 300 mg/L of methylene blue solution. Five solutions of methylene blue at 2.4 mg/L, 1.2 mg/L, 0.60 mg/L, 0.48 mg/L, and 0.12 mg/L were prepared. The absorbances of the above five concentrations of methylene blue solution were determined using an ultraviolet spectrophotometer and the standard curve was plotted as Figure 1, with the test wavelength set at 665 nm. Additionally, 20 mg of activated carbon samples were weighed and dissolved in 100 mL of methylene blue solution at a concentration of 300 mg/L and then transferred to conical flasks for two parallel experiments for each activated carbon sample. The conical flask was placed in a water bath thermostatic shaker for 20 min at 25 °C. The samples were filtered through a medium-speed qualitative filter paper and then diluted with buffer solution to a concentration range of 0.12–2.4 mg/L and loaded into the flask for measurement.

## 3. Results and Discussion

### 3.1. Specific Surface Area and Pore Size Distribution Analysis

**Figure 2:**
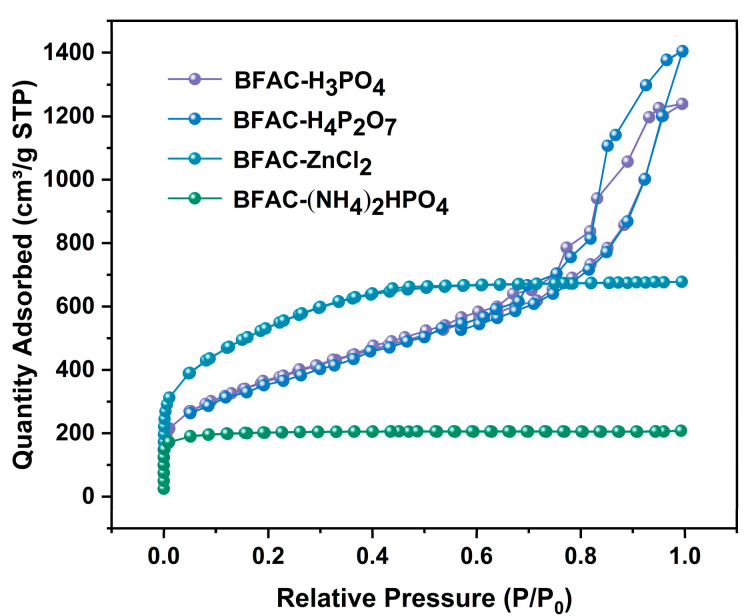
Nitrogen adsorption/desorption isotherms of BFACs activated by different activators.

**Figure 3:**
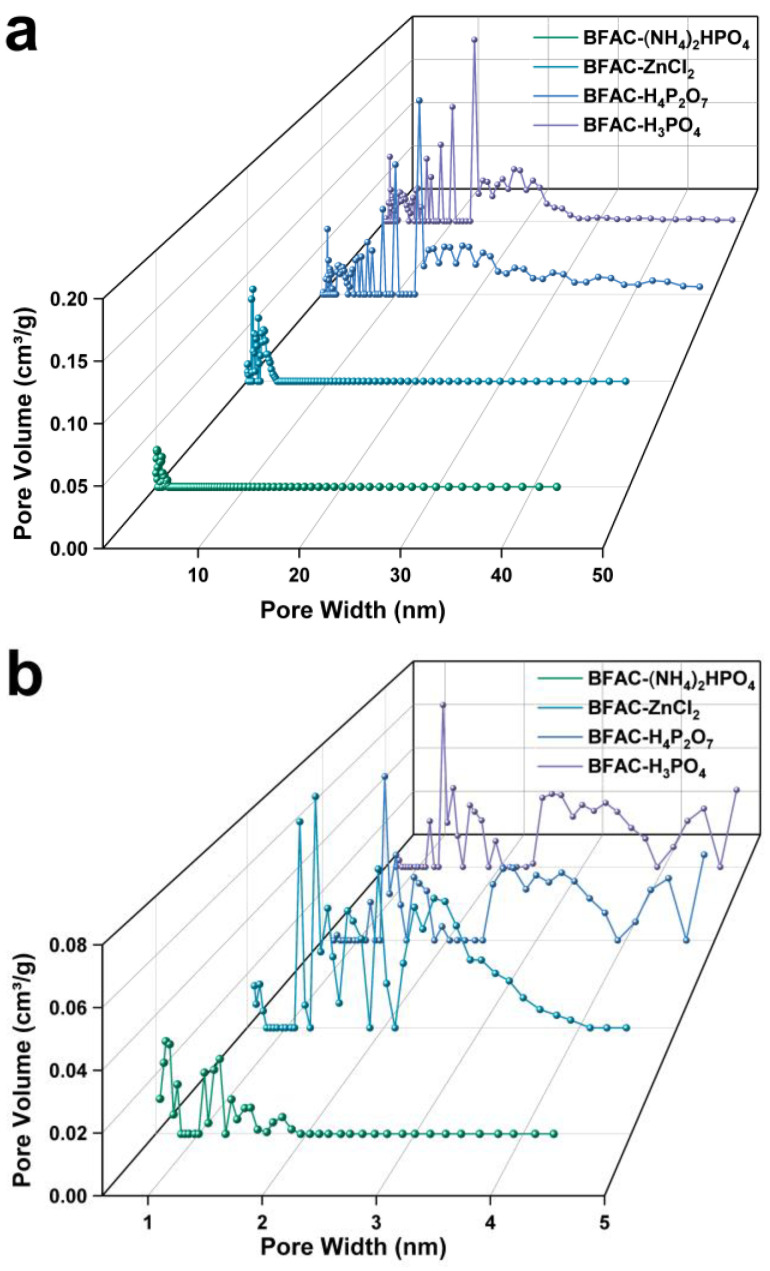
Pore size distribution curves of BFACs activated by different activators: (**a**) mesoporous; (**b**) micropores.

The specific surface area and pore size distribution of BFAC are one of the effective indicators to characterize its adsorption performance [17]. From Figure 2, the initial segment of the isotherm of the four samples showed a significantly large and steep rise when the relative pressure was less than 0.01. After the relative pressure was greater than 0.01, the adsorption of BFAC-H_3_PO_4_ and BFAC-H_4_P_2_O_7_ gradually increased with the relative pressure, which was mainly multilayer adsorption. Additionally, the curves showed an upward curve at a higher relative pressure, which belonged to capillary coalescence phenomenon, and the adsorption isotherms belonged to combined type II and type IV according to IUPAC classification and there was an obvious H2-type hysteresis loop at the end of the curve, indicating that these two samples belonged to ink bottle-shaped mesoporous materials [18]; the pore structure of BFAC-H_4_P_2_O_7_ was closer to the ink bottle-shaped. The nitrogen adsorption and desorption curves of BFAC-(NH_4_)_2_HPO_4_ belonged to the typical type I. The entire adsorption platform was in a horizontal straight line after the relative pressure is greater than 0.1, indicating that the activated carbon was a microporous material; the data in Table 1 confirmed this conclusion [19]. The curve of BFAC-ZnCl_2_ showed a knee-like bending change near the relative pressure of 0.1, mainly because more and more nitrogen molecules were introduced into the system, which was also related to the specific surface area and pore size distribution of the micropores of BFAC-ZnCl_2_ (as shown in Table 1 and Figure 3, indicating that the sample had a relatively wide pore size distribution [20].

From Table 1, the BET-specific surface area of activated carbon prepared by ZnCl_2_ activation was the largest among all the samples (1908.51 m^2^/g), illustrating that ZnCl_2_ had the potential to prepare activated carbon with a high specific surface area. The specific microporous surface area of BFAC-(NH_4_)_2_HPO_4_ was 613.34 m^2^/g, about 95.6% of the total specific surface area, showing that (NH_4_)_2_HPO_4_ was suitable for the preparation of microporous activated carbon. The mesoporous specific surface areas of BFAC-H_3_PO_4_ and BFAC-H_4_P_2_O_7_ were 900.50 and 876.58 m^2^/g, accounting for 68.3% and 69.3% of the total specific surface area, respectively, which had a richer mesoporous structure compared with BFAC-ZnCl_2_ and BFAC-(NH_4_)_2_HPO_4_. It was explained that the preparation of activated carbon by the phosphoric acid method was more suitable for use as a mesoporous adsorbent, whose adsorbent mechanism was capillary condensation [21]. According to Figure 3a, the samples of pyrophosphate and phosphoric acid pyrophosphate had a broader and similar pore size distribution. In contrast, BFAC-ZnCl_2_ and BFAC-(NH_4_)_2_HPO_4_ were mainly microporous. Figure 3b shows more clearly the pore size distribution of micropores and micromesopores and BFAC-H_3_PO_4_ and BFAC-H_4_P_2_O_7_ were basically the same. BFAC-ZnCl_2_ had well-developed micropores and was mainly concentrated below 4 nm. BFAC-(NH_4_)_2_HPO_4_ had the narrowest pore size distribution and the smallest pore size (<2 nm) and pore volume (<0.04 cm^3^/g). Consistent with Table 1, the micropore structure of BFAC-ZnCl_2_ has a maximum pore size of 1.3 nm, followed by 1.1 nm and 1.9 nm and there are also some micropores with pore sizes in the range of 1.5 nm. It indicates that the pore size distribution of BFAC-(NH_4_)_2_HPO_4_ was more uniform. 

### 3.2. FT-IR Analysis

**Figure 4:**
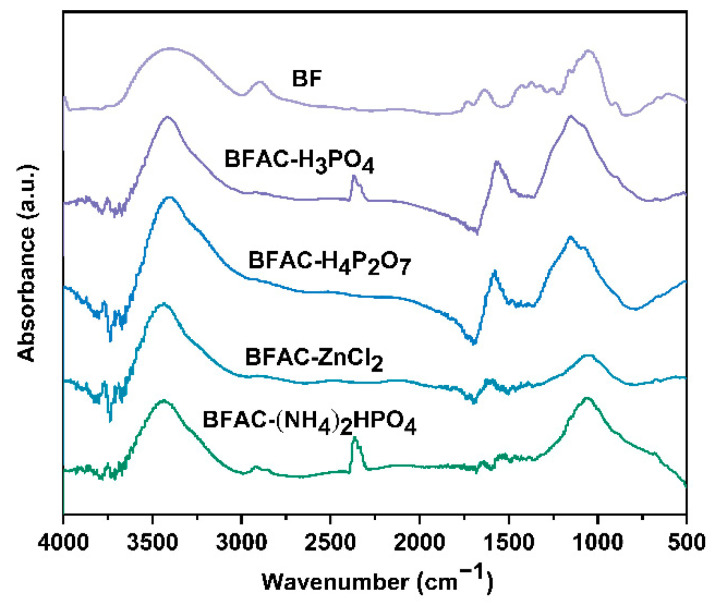
FT-IR spectra of the BFACs activated by different activators and BF.

The FT-IR spectra of the four BFACs and BF were shown in Figure 4. The spectra showed a strong absorption peak at 3400 cm^−1^ associated with the O–H stretching vibration, which increased in intensity after activation, indicating an increase in the -OH content. The absorption peak in the region (4000–3750 cm^−1^) was caused by the absorption of water molecules by the activated carbon sample [22]. The absorption peak observed at 2920 cm^−1^ and 2850 cm^−1^ was associated with the stretching vibration of the -CH_2_- group. BF had a cellulose -CH_2_- functional group, so it had a strong peak here. After activation by (NH_4_)_2_HPO_4_, some of the -CH_2_- groups remained. In contrast, the weak peak of the other sample indicated that a small number of saturated hydrocarbons are present in the other sample and that most of the -OCH_3_ groups attached to the aromatic ring have been dissociated during activation [23]. The band at 1620 cm^−1^ can be ascribed to C–C aromatic ring stretching vibration [24]; this band of BFAC-H_3_PO_4_ and BFAC-H_4_P_2_O_7_ was much higher than BFAC-ZnCl_2_ and BFAC-(NH_4_)_2_HPO_4_, indicating that phosphoric acid and pyrophosphate have a certain aromatization effect. The strong peak at 1080 cm^−1^ indicates the presence of alcohol groups, which indicates the presence of polysaccharides [25].

### 3.3. X-ray Diffraction Analysis

**Figure 5:**
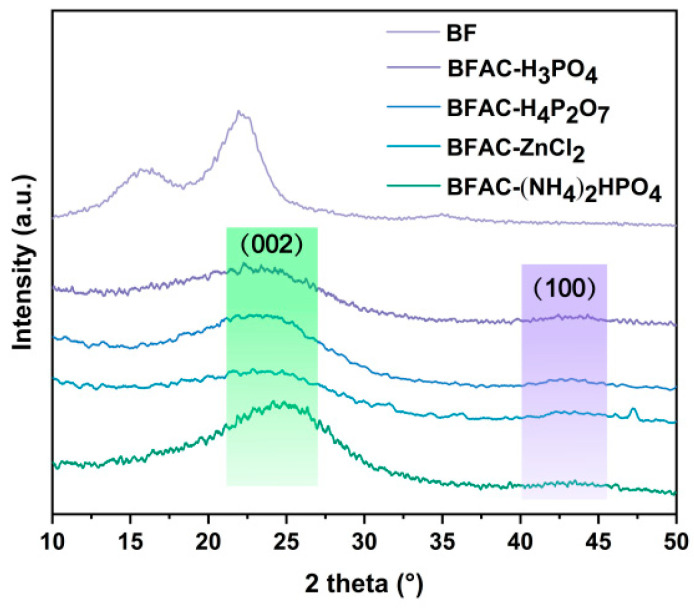
X-ray diffraction patterns of BFACs activated by different activators and BF.

The prominent diffraction peaks of BF were around 15.8°, 22.76°, and 34.7°, which were consistent with the crystallographic planes of (110), (200), and (004), respectively [26]. The XRD pattern of BFACs in Figure 5 revealed characteristic diffraction peaks (2θ = 23° and 43°) that correspond to the (002) and (100) signatures, respectively [27]. Additionally, the existence of broad XRD peaks in the spectrum indicated the amorphous nature of the BFACs’ structure. This characteristic is beneficial for the adsorption behavior material, because gas or liquid generally penetrated the cellulose matrix through the amorphous phase [28]. BFAC-(NH_4_)_2_HPO_4_ showed a higher intensity (002) crystallographic surface diffraction characteristic peak compared with the other three groups. It can be seen from Table 2 that the 2θ_002_ of the four samples were 23.0, 22.5, 23.0, and 24.3 and the crystal plane spacing d_002_ was 0.386, 0.395, 0.372, and 0.366, respectively. BFAC-(NH_4_)_2_HPO_4_ had the smallest d002 value, which indicates that this activated carbon sample has a higher degree of graphitization and a more ordered internal structure, indicating that the activation of diammonium impregnation is more favorable to the formation of graphitic microcrystalline structure in activated carbon. Therefore, the adsorption capacity of the BFAC-(NH_4_)_2_HPO_4_ might be poor.

### 3.4. XPS Analysis

**Figure 6:**
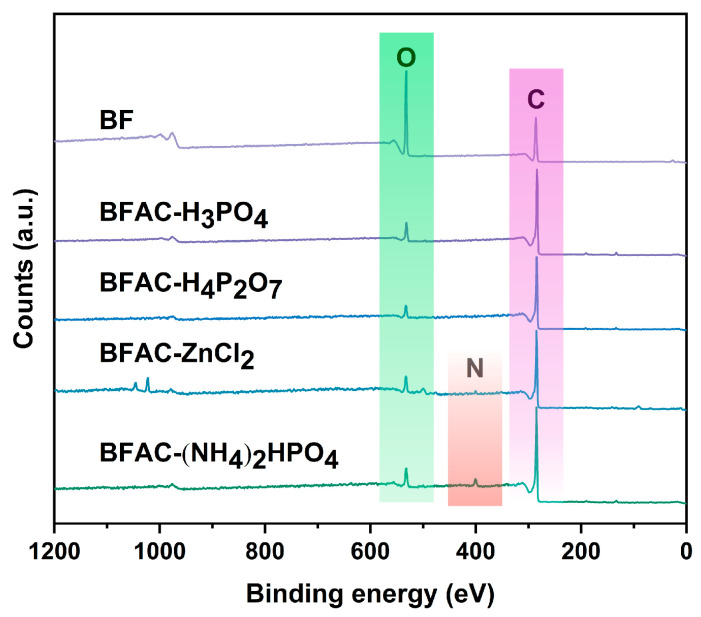
XPS spectra of BFACs activated by different activators and BF.

Figure 6 showed the XPS spectra of the BFACs and BF, which indicated that the BFACs contained the elements C (284 eV), O (532 eV), and N (398 eV). The N1s XPS peak was not found in the spectra of BFAC-H_3_PO_4_ and BFAC-H_4_P_2_O_7_, but the (NH_4_)_2_HPO_4_ activation brings nitrogen-containing functional groups to BF, resulting in the appearance of the N active group.

**Figure 7:**
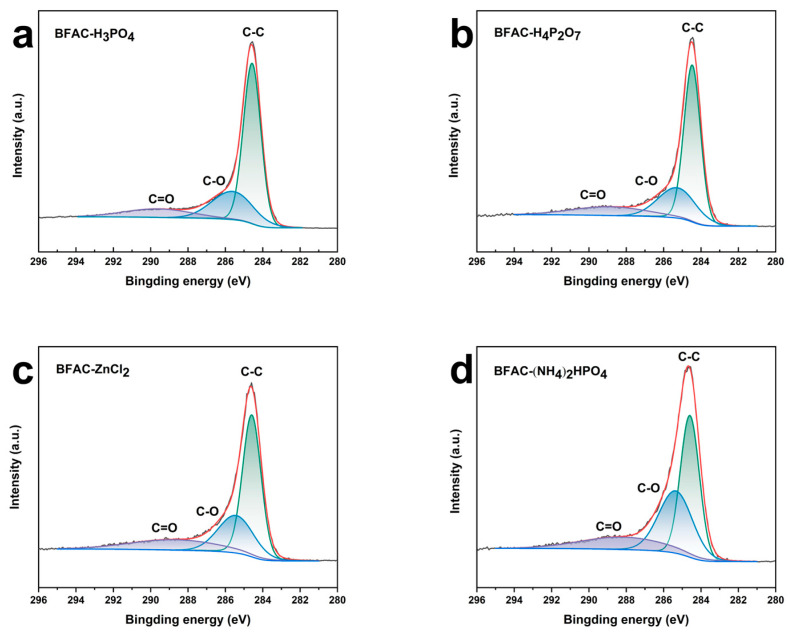
C1s spectra of BFACs activated by different activators (**a**) BFAC-H_3_PO_4_, (**b**) BFAC-H_4_P_2_O_7_, (**c**) BFAC-ZnCl_2_, (**d**) (NH_4_)_2_HPO_4_.

**Figure 8:**
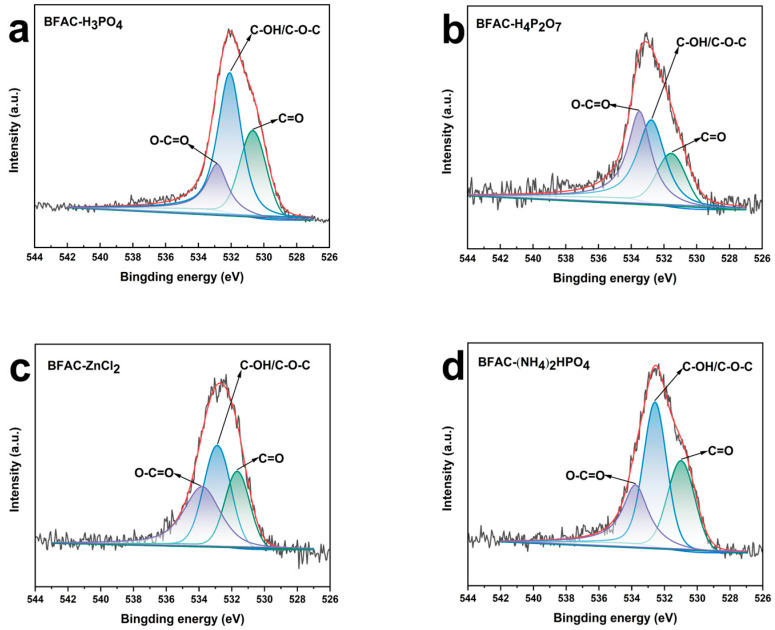
O1s spectra of BFACs activated by different activators (**a**) BFAC-H_3_PO_4_, (**b**) BFAC-H_4_P_2_O_7_, (**c**) BFAC-ZnCl_2_, (**d**) (NH_4_)_2_HPO_4_.

The C1s spectra for BFACs samples from Figure 7 and Figure S1 mainly displayed three peaks corresponding to the C=O (oxygen doubly bonded to aromatic carbon), C–O (oxygen singly bonded to aliphatic carbon), and C–C [29]. In addition, the O1s spectra for BFACs samples from Figure 8 and Figure S2 displayed mainly three peaks corresponding to the O–C=O, C–OH/C–O–C, and C=O. Figure 7 showed the C1s spectra of BFACs activated by different activators. BFAC-H_3_PO_4_ (Figure 7a), BFAC-H_4_P_2_O_7_ (Figure 7b), and BFAC-ZnCl_2_ (Figure 7c) had a similar C1s range, explaining the carbon and oxygen content was identical. However, the contents of oxygen-containing functional groups were not identical in the three samples. Compared to BFAC-H_3_PO_4_ (Figure 8a), BFAC-H_4_P_2_O_7_ (Figure 8b) had a lower C=O content, while BFAC-ZnCl_2_ (Figure 8c) had a lower C–OH/C–O–C, and a higher O–C=O content. The highest C–O was found in the (NH_4_)_2_HPO_4_ activated samples (Figure 7d). This was mainly due to the contribution of C–OH/C–O–C bonds (Figure 8d). The large oxygen-containing functional groups on the sample’s surface could improve the electrochemical wettability and dye adsorption properties [30].

### 3.5. Electrochemical Performance Analysis

**Figure 9:**
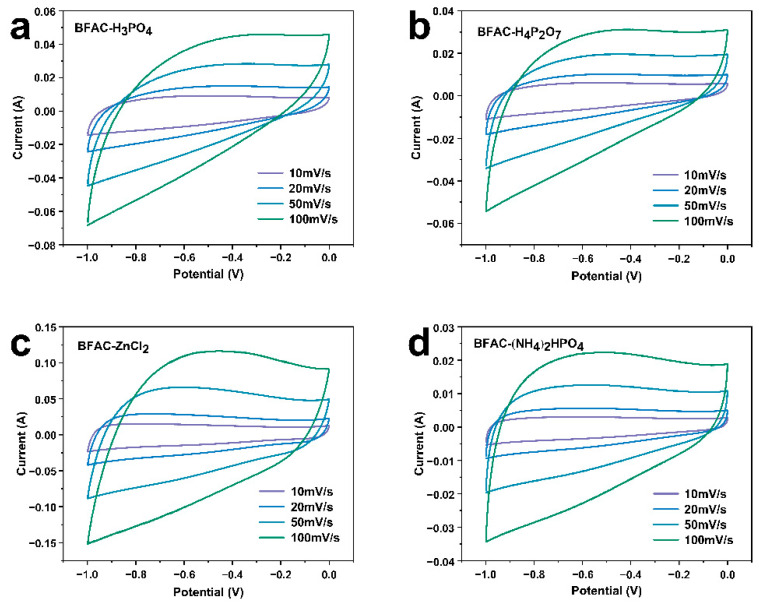
CV comparison of BFACs at from 10 to 100 mV/s scan rates (**a**) BFAC-H_3_PO_4_, (**b**) BFAC-H_4_P_2_O_7_, (**c**) BFAC-ZnCl_2_, (**d**) (NH_4_)_2_HPO_4_.

The effects of bamboo fiber-based activated carbon by different activators on the capacitive characteristics in a 6 M KOH electrolyte were investigated using a three-electrode system. The electrochemical energy storage performance of BFACs was assessed by CV measurements and the linear sweep rates set in this experiment are 10, 20, 50, and 100 mV/s (Figure 9). With the increase in scan rates from 10 to 100 mV/s, the current densities and area under the forward and reverse scan of the CV curves increased. This trend is because, at slower scan rates, electrolyte ions were entirely diffused into the electrode materials [31]. Four BFACs samples, having a similar shape of a CV curve at a scan rate of 10 mV/s, which exhibits a quasi-rectangular shape, showed good electrochemical properties. However, the four BFACs samples exhibit different electrochemical properties at a scan rate of 100 mV/s. The CV curve of BFAC-H_3_PO_4_ showed fusiform, indicating electrolyte diffusion is difficult. The other three samples presented the rectangle at a scan rate of 100 mV/s, illustrating excellent reversibility for electrolyte ions to adsorb/desorb among the surface of the electrode materials and the incredible rate performance [32]. BFAC-ZnCl_2_ had the most significant current because it had the largest specific surface area and the wealthiest pore structure, from which it could be inferred that BFAC-ZnCl_2_ had the most prominent specific capacitance. The pore structures of BFAC-H_3_PO_4_ and BFAC-H_4_P_2_O_7_ were mainly mesoporous, but the shape of the CV curve at a scan rate of 100 mV/s was different; it is speculated that it was related to the pore shape. The electrolyte diffusion rate was related to the shape of the pore. The shape of BFAC-H_4_P_2_O_7_ was closer to the rectangle, so the electrolyte was spreading faster at a high scanning rate.

**Figure 10:**
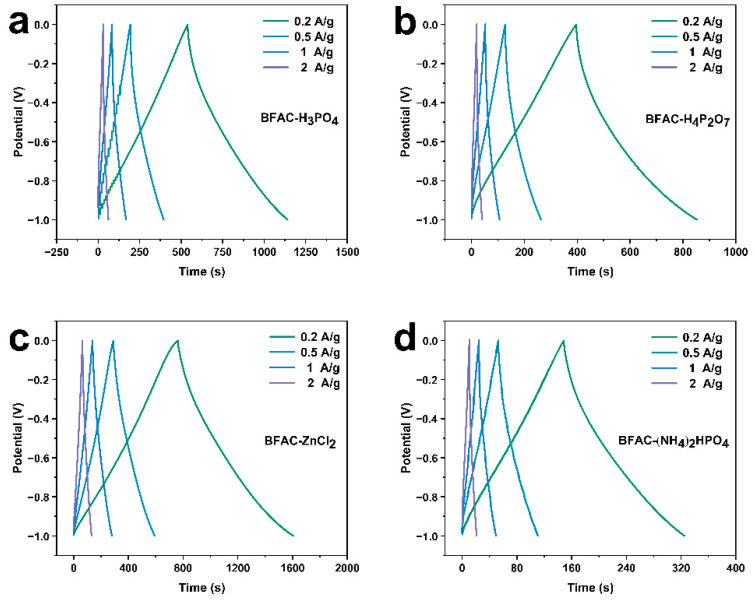
Charge/discharge curves of BFACs at various current densities (**a**) BFAC-H_3_PO_4_, (**b**) BFAC-H_4_P_2_O_7_, (**c**) BFAC-ZnCl_2_, (**d**) (NH_4_)_2_HPO_4_.

The GCD curves of BFACs over 0.2, 0.5, 1, and 2 A/g current densities were displayed in Figure 10. The charge/discharge graphs of four BFACs samples revealed symmetrical triangular shapes at various current densities, demonstrating its electrical double layer capacitors (EDLCs) behavior and excellent reversibility [33]. The GCD figures were presented in Table 3. Compared with other samples, BFAC-ZnCl_2_ exhibited the longest charging (760.7 s) and discharging times (844.93 s), calculating that the BFAC-ZnCl_2_ electrode had the maximum specific capacitance of 169.79 F/g at 0.2A/g. As the reasonable pore structure distribution and maximum specific surface area of the BFAC-ZnCl_2_ electrode enhanced the acceptance of K+ and OH− hydrated ions, the performance of the BFAC-ZnCl_2_ electrode materials was the most excellent compared to the other samples [34]. 

**Figure 11:**
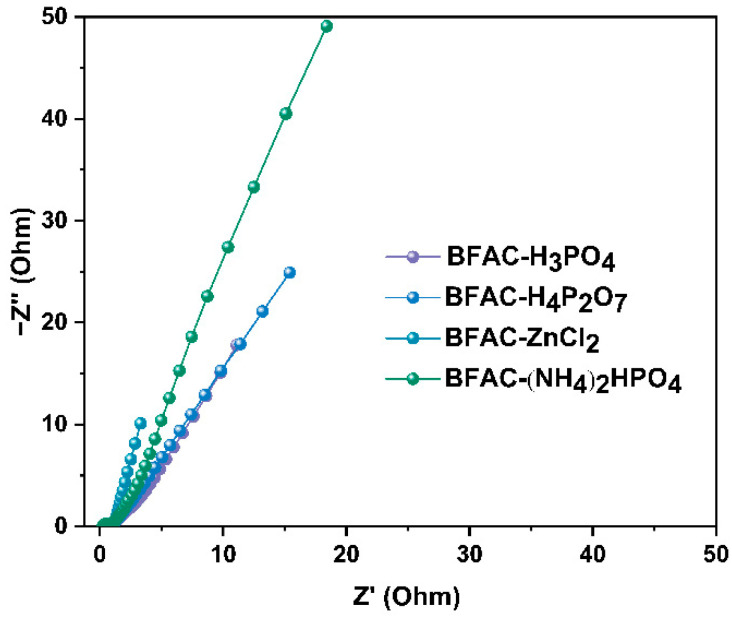
Electrochemical impedance spectroscopy of BFACs.

In order to understand the ion and electron transport of the BFAC electrodes in 6M KOH electrolyte, electrochemical impedance spectroscopy (EIS) measurements were performed and the results were shown in Figure 11. The Nyquist plots of all the samples exhibited a linear trend at a low frequency, indicating an ideal capacitive behavior of the electrodes [35]. Compared to the other samples, the Nyquist plots of BFAC-ZnCl_2_ was closer to vertical in the low-frequency region, demonstrating better capacitance characteristics and faster ion diffusion rates about BFAC-ZnCl_2_. At a high frequency, series resistance (Rs) was obtained from the intercept at the real axis (Z’) in the Nyquist plot; the Rs of BFAC-ZnCl_2_ is the lowest [36].

### 3.6. Adsorption Performance Analysis

In previous studies, f single molecular layers, as a porous adsorption material, in addition to the surface chemical composition of dye adsorption, a pore structure, also had a certain impact on adsorption.

As can be seen from Table 4, the adsorption performance of BFAC-ZnCl_2_ is the best (648.75 mg/g); this sample contains more micropores with larger pore sizes and smaller pore size mesopores, indicating that the content of larger micropores and mesopores plays a major role in the adsorption of activated carbon fibers to methylene blue. In addition, compared to BFAC-H_3_PO_4_ (373.33 mg/g), BFAC- H_4_P_2_O_7_ of similar void distribution, crystal form, and chemical composition had a higher MB adsorption capacity (548.75 mg/g), which proved that the ink bottle-shaped pore structure was advantageous to the adsorption of MB. BFAC-(NH_4_)_2_HPO_4_ had the lowest amount of MB adsorption (218.75 mg/g) due to the lowest specific surface area mostly correlating with adsorption capacity.

## 4. Conclusions

In this work, the effects of four activators, namely phosphoric acid, pyrophosphoric acid, zinc chloride, and ammonium dihydrogen phosphate, on the structure and properties of bamboo fiber cell-based activated carbon were investigated. As previously shown, BFAC-(NH_4_)_2_HPO_4_ had the smallest BET specific surface area and the pore structure was dominated by micropores, with the pore size distribution concentrated from 0.5 nm to 0.8 nm and from 1 nm to 1.5 nm. On the contrary, BFAC-ZnCl_2_ has the highest specific surface area and the pore structure was mainly micropores and mesopores, with the pore size distribution concentrated from 1 to 2 nm and from 2.1 to 4 nm, respectively. In contrast, BFAC-H_3_PO_4_ and BFAC-H_4_P_2_O_7_ have moderate specific surface areas, with basically similar pore size distributions and the pore structures are mainly mesoporous. 

In terms of electrochemistry, the specific capacitance of BFAC-ZnCl_2_ was the largest. At the current density of 0.2 A/g, the specific capacitance was 121.2730 F/g. Additionally, the internal resistance of the sample was small, which reflects the good electrochemical reversibility and capacitance performance. From the perspective of the adsorption performance, the adsorption amount of methylene blue is affected by the BET specific surface area, pore volume, surface functional group content, and other factors. Considering various factors, BFAC-ZnCl_2_ has the best adsorption performance. The superior adsorption capacity of activated carbon has the potential for applications in catalysis, drug loading, and heavy metal enrichment.

## Figures and Tables

**Table 1:** Specific surface area BET and pore structure parameters of BFACs.

Sample	S_BET_ (m^2^/g)	S_mic_ (m^2^/g)	S_mes_ (m^2^/g)	V_tot_ (cm^3^/g)	Micro- Porosity (%)	Meso- Porosity (%)	Average Pore Size (nm)
BFAC-H_3_PO_4_	1318.06	240.77	900.50	1.92	5.12	85.94	7.32
BFAC-H_4_P_2_O_7_	1264.14	207.89	876.58	2.17	3.91	76.34	7.57
BFAC-ZnCl_2_	1908.51	1092.21	761.39	1.05	44.99	48.61	2.20
BFAC- (NH_4_)_2_HPO_4_	641.59	613.34	22.29	0.32	92.95	5.47	2.00

**Table 2:** BFACs crystal structure parameters. (L_c_) Gaphite accumulation height, (L_c_/d_002_) number of graphite stacking layers.

Sample	2θ_002_ (°)	2θ_100_ (°)	d_002_ (nm)	L_c_ (nm)	L_a_ (nm)	Lc/d002
BFAC-H_3_PO_4_	23.0	43.2	0.386	0.685	3.175	1.773451
BFAC-H_4_P_2_O_7_	22.5	43.3	0.395	0.793	3.425	2.008551
BFAC-ZnCl_2_	23.0	43.4	0.372	0.764	3.013	2.055704
BFAC-(NH_4_)_2_HPO_4_	24.3	43.5	0.366	0.883	3.121	2.412869

**Table 3:** Charge and discharge time and specific capacitance of BFACs.

Sample	Current Density (A/g)	Charging Time (s)	Discharge Time (s)	Specific Capacitance (F/g)
BFAC- H_3_PO_4_	0.2	534.20	600.67	121.2730
	0.5	190.53	201.13	102.9170
	1	81.633	84.033	87.9411
	2	31.100	31.433	68.8513
BFAC- H_4_P_2_O_7_	0.2	396.73	456.47	92.2130
	0.5	127.77	136.07	69.6466
	1	52.300	54.167	56.6848
	2	19.967	20.167	44.0601
BFAC- ZnCl_2_	0.2	760.70	844.93	169.7910
	0.5	290.70	302.17	152.8050
	1	138.67	141.37	144.4940
	2	65.133	65.733	137.2640
BFAC- (NH_4_)_2_HPO_4_	0.2	148.80	176.2	35.6055
	0.5	54.167	57.833	29.6374
	1	24.200	24.933	26.1562
	2	10.567	10.567	23.1743

**Table 4:** Methylene blue adsorption of BFACs.

Sample	MB Initial Concentration (mg/L)	MB Termination Concentration (mg/L)	Adsorption Capacity (mg/g)	Termination Absorbance
BFAC- H_3_PO_4_	300	225.33	373.33	0.133
BFAC- H_4_P_2_O_7_	300	190.25	548.75	0.113
BFAC- ZnCl_2_	300	170.25	648.75	0.101
BFAC- (NH_4_)_2_HPO_4_	300	256.25	218.75	0.151

## Data Availability

Not applicable.

## Supplementary Materials

The following supporting information can be downloaded at: https://www.mdpi.com/article/10.3390/polym14245500/s1, Figure S1: C1s spectra of BF; Figure S2: O1s spectra of BF.
