# Supplementary material for: Influence of Different Activators on the Structure and Properties of Activated Carbon Based on Bamboo Fiber
Source: Polymers (Basel). 2022 Dec 15;14(24):5500. doi: 10.3390/polym14245500 (PMC9788235; doi:10.3390/polym14245500)
Supplement: Supplementary file 1 [file polymers-14-05500-s001.zip › polymers-2084430-supplementary/Supplementary Material.pdf]

Supplementary Information

Influence of Different Activators on the Structure and Properties of Activated Carbon Based on Bamboo Fiber

Peng Lin, Yao Xia, Zhigao Liu*

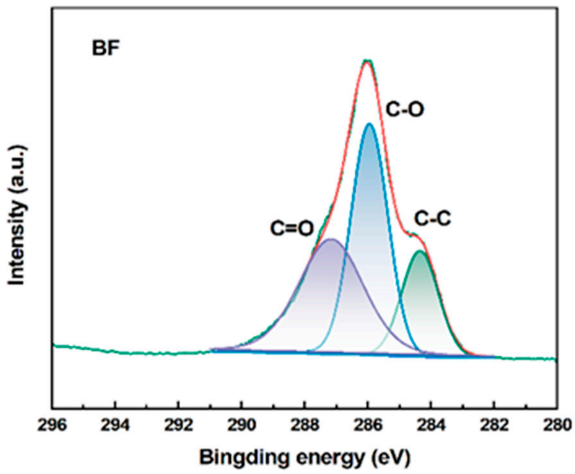

Figure S1. C1s spectra of BF

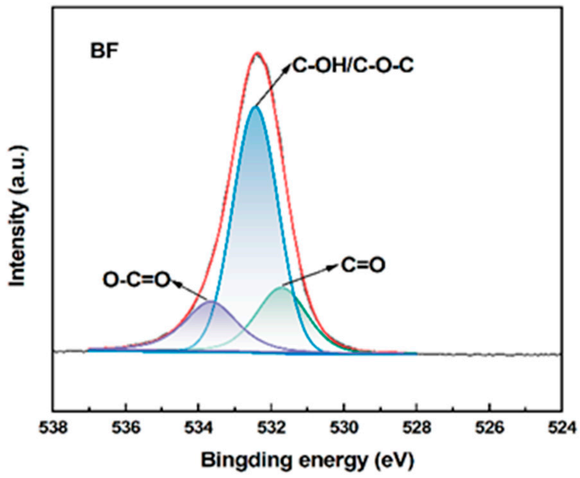

Figure S2. spectra of BF
